# Novel Estrogen Receptor Dimerization BRET-Based Biosensors for Screening Estrogenic Endocrine-Disrupting Chemicals

**DOI:** 10.34133/bmr.0010

**Published:** 2024-03-07

**Authors:** Gyuho Choi, Hyunkoo Kang, Jung-Soo Suh, Haksoo Lee, Kiseok Han, Gaeun Yoo, Hyejin Jo, Yeong Min Shin, Tae-Jin Kim, BuHyun Youn

**Affiliations:** ^1^ Department of Integrated Biological Science, Pusan National University, Busan 46241, Republic of Korea.; ^2^ Food Safety Risk Assessment Division, National Institute of Food and Drug Safety Evaluation, Ministry of Food and Drug Safety, Cheongju 28159, Republic of Korea.; ^3^ Department of Biological Sciences, Pusan National University, Busan 46241, Republic of Korea.; ^4^ Institute of Systems Biology, Pusan National University, Busan 46241, Republic of Korea.; ^5^ Nuclear Science Research Institute, Pusan National University, Busan 46241, Republic of Korea.

## Abstract

The increasing prevalence of endocrine-disrupting chemicals (EDCs) in our environment is a growing concern, with numerous studies highlighting their adverse effects on the human endocrine system. Among the EDCs, estrogenic endocrine-disrupting chemicals (eEDCs) are exogenous compounds that perturb estrogenic hormone function by interfering with estrogen receptor (ER) homo (α/α, β/β) or hetero (α/β) dimerization. To date, a comprehensive screening approach for eEDCs affecting all ER dimer forms in live cells is lacking. Here, we developed ER dimerization-detecting biosensors (ERDDBs), based on bioluminescence resonance energy transfer, for dimerization detection and rapid eEDC identification. To enhance the performance of these biosensors, we determined optimal donor and acceptor locations using computational analysis. Additionally, employing HaloTag as the acceptor and incorporating the P2A peptide as a linker yielded the highest sensitivity among the prototypes. We also established stable cell lines to screen potential ER dimerization inducers among estrogen analogs (EAs). The EAs were categorized through cross-comparison of ER dimer responses, utilizing EC values derived from a standard curve established with 17β-estradiol. We successfully classified 26 of 72 EAs, identifying which ER dimerization types they induce. Overall, our study underscores the effectiveness of the optimized ERDDB for detecting ER dimerization and its applicability in screening and identifying eEDCs.

## Introduction

Endocrine-disrupting chemicals (EDCs) are chemical substances that disrupt the endocrine system and exert adverse effects on both the environment and living organisms. EDCs can infiltrate the human body through various routes, including inhalation, dermal absorption, and dietary intake, leading to their accumulation over prolonged periods [1]. Recent studies have highlighted the profound impact of EDCs on a range of biological processes, including fetal development, reproduction, metabolism, and immune function, making them a significant public health concern [2,3]. The ubiquitous presence of specific EDCs such as bisphenol A, commonly found in plastics and consumer products, exemplifies the urgent need for effective monitoring and regulatory measures to mitigate their pervasive impact on the endocrine system [4]. Among the diverse array of EDCs, estrogenic EDCs (eEDCs) stand out for their ability to mimic or interfere with the actions of the female hormone, 17β-estradiol (E2), resulting in disruptions within the estrogenic endocrine system [5]. Our understanding of potential eEDCs continues to expand; however, accurately evaluating their endocrine-disrupting activity remains a complex challenge. A rapid and reliable screening assay is urgently needed to safeguard human health and the environment.

Estrogen receptor (ER) signaling encompasses three key steps: ligand-receptor binding, subsequent ER dimerization, and ER dimer interaction with estrogen response elements (EREs), leading to ERE-mediated transcriptional activation [6]. eEDCs can directly affect ER dimerization by interacting with the ER or indirectly modulate ERE-mediated transcription by altering E2 metabolism or activating other signaling pathways [7]. Direct-acting EDCs disrupt estrogen signaling mediated by two distinct ERs, ERα and ERβ, both of which function as ligand-activated transcription factors within the nuclear receptor superfamily. These ERs exhibit varying expression patterns, subcellular localization, and transcriptional activities depending on the cellular context and the ligand in use [8]. In response to ligand binding, ERα and ERβ can form homodimers (ERα/ERα or ERβ/ERβ) or heterodimers (ERα/ERβ), and both dimer types can bind EREs [9,10]. Each ER dimer type has distinct roles in cellular processes. ERα homodimers are known to drive breast cancer growth, while ERβ homodimers exhibit anti-proliferative effects [11]. The heterodimers, consisting of both ERα and ERβ, provide a unique balance in regulating gene expression and cellular functions [12]. Since different EDCs induce varying dimerization patterns based on their specific characteristics, it is imperative to develop methods for evaluating potential EDCs with respect to each dimer type.

The Organization for Economic Co-Operation and Development (OECD) offers two essential in vitro test guidelines, the ER binding (TG 493) [13] and ER transactivation (TG 455) [14] assays, for evaluating the potential hazards and risks associated with eEDCs. The ER binding assay assesses the binding capacity of [^3^H] E2 to ERs in the presence of a test substance, thus gauging ligand-receptor binding. Meanwhile, the ER transactivation assay measures human ERα-mediated ERE-reporter activity, providing insights into ERE-mediated transcriptional activation. Although these methods are valuable, developing test guidelines that address all three ER signaling pathways for the comprehensive evaluation of potential eEDCs is crucial. Unfortunately, the OECD has yet to adopt any test guidelines that specifically assess ER dimerization.

In this study, we developed bioluminescence resonance energy transfer (BRET)-based biosensors designed to detect ER dimerization along with stable cell lines expressing these biosensors in both homodimeric and heterodimeric configurations. The BRET method is widely utilized in screening assays, primarily because of its diminished vulnerability to interference from cell autofluorescence and photobleaching of the donor and acceptor. This advantage stems from the low light emission characteristic of luciferase [15,16]. We optimized these biosensors by identifying the ideal positions for tagging donor and acceptor proteins onto the ER as well as the appropriate acceptor proteins and protein linkers, all of which maximize sensitivity. The newly developed ER dimerization-detecting biosensors (ERDDBs) offer a rapid and precise means of identifying eEDCs, thereby enhancing our capacity to assess their effects.

## Materials and Methods

### Cell culture

Lenti-X 293T (Clontech, 632180) and MCF-7 (Korea Cell Line Bank, 30022) cells were cultured in Dulbecco’s modified Eagle’s medium (DMEM; GenDepot, CM-002) supplemented with 10% fetal bovine serum (FBS; Gibco, 16000-044) and 100 U/ml penicillin + 100 μg/ml streptomycin (GenDepot, CA005). The cells were grown in a humidified incubator containing 5% CO_2_ at 37 °C.

### Computational structure prediction

The structures of the ERα and ERβ ligand binding domain (LBD) dimers were obtained from Protein Data Bank (PDB) (PDB ID: 5wgd and 5toa, respectively). The full lengths of the ERα and ERβ monomers were obtained from the alpha fold protein structure database (AFDB) (AFDB code: AF-P03372 and AF-Q92731, respectively). To rearrange the ER LBD dimer and ER full-length monomer, we used an alignment tool in PyMOL.

### Plasmid construction

ERDDB was constructed by amplifying each domain using polymerase chain reaction (PCR), digestion, and ligation, according to the sequence shown in Fig. 2E. CyOFP1 and HaloTag were amplified from pKK-TEV-CyOFP1 (Addgene #105799) and pHAGE-EFS-MCP-HALOnls (Addgene #121937), respectively, and digested using BamHI and XhoI. The EV linker and P2A peptide sequences were amplified from pGEMT-TPE2A-Mef2c-Tdtomato-Gata4-Tbx5 (Addgene #111818) and pCAGGS-6011nes (Addgene #108652), respectively, and then digested with BspEI/MluI for the α/α and α/β ERDDB or BspEI/NotI for the β/β ERDDB. Nano-luciferase was amplified from pcDNA3.1-CAG-NanoLuc-VChR1-EYFP (Addgene #114112) and digested with NotI/XbaI. ERα and ERβ were amplified from pEGFP-C1-ER alpha (Addgene #28230) and pEGFP-C1-ER beta (Addgene #28237), respectively. For the α/α and α/β ERDDBs, the amplified construct was digested with XhoI/BspEI or XbaI/PspOMI, respectively, and for β/β ERDDB, with XhoI/BspEI or MluI/NotI. For lentiviral production, all ERDDBs were digested with BamHI/PspOMI and ligated into the pLenti-puro vector (Addgene #39481).

### Chemicals and drugs

Transfections were performed using PEIpro (Polyplus, 115-010) according to the manufacturer’s instructions. E2 (S1709), 4-hydroxytamoxifen (S7827), H3B-5942 (S8746), fulvestrant (S1191), diethylstilbestrol (S1859), dienestrol (S1858), α-estradiol (S5910), and corticosterone (S4752) were obtained from Selleck Chem. Angolensin (SMB01023) was purchased from Sigma-Aldrich, and 72 EAs were obtained from the Ministry of Food and Drug Safety of the Republic of Korea. Each drug and EA was diluted in dimethyl sulfoxide (DMSO). DMSO was purchased from Biosesang (AC4002-050-00, Seongnam, Republic of Korea). For the BRET assay, HaloTag NanoBRET 618 Ligand (G980A), NanoBRET Nano-Glo Substrate (N157A), and Nano-Glo Endurazine Substrate (N257B) were purchased from Promega. For imaging, Hoechst 33342 (H1399) was purchased from Invitrogen.

### Western blots

Protein expression was validated as previously described [17]. Briefly, whole-cell lysates were prepared using radioimmunoprecipitation assay (RIPA) lysis buffer [50 mM tris, pH 7.4, 150 mM NaCl, 1% Triton X-100, 25 mM NaF, 1 mM dithiothreitol, and 20 mM ethylene glycol tetraacetic acid supplemented with protease inhibitors (Thermo Fisher Scientific, 78429)], and protein concentrations were determined using the Bio-Rad protein assay dye reagent concentrate (Bio-Rad Laboratories, 5000006). Protein samples were subjected to 8% sodium dodecyl sulfate–polyacrylamide gel electrophoresis, transferred to a nitrocellulose membrane, and blocked with 5% bovine serum albumin in tris-buffered saline containing Tween 20 (10 mM tris, 100 mM NaCl, and 0.1% Tween 20) for 1 h. The membranes were incubated with primary antibodies against ERα (Abcam, ab32063), ERβ (Abcam, ab288), and α-tubulin (Invitrogen, 62204) for 1.5 h, followed by incubation with peroxidase-conjugated anti-mouse and anti-rabbit IgG secondary antibodies (Thermo Fisher Scientific, 31430, 31460, respectively) for 1 h. Blots were imaged using an ECL detection system (Thermo Fisher Scientific, 34580) with an iBright FL1000 Imaging System (Thermo Fisher Scientific, A32748).

### BRET assay and measurements

For the optimization of the acceptor and linker, ERα/β BRET biosensors were transfected in the HEK-293T cell line. The cells were harvested 24 to 36 h after transfection and resuspended in assay media. Then, 100,000 cells were added to a white-bottom 96-well plate (SPL, 30196) and incubated overnight. E2 was added to the wells at a 1 μM final concentration. After 24-h treatment, NanoBRET Nano-Glo Substrate (final dilution 1:200) was injected into wells 15 min before measuring. Luminescence signals were read in a CLARIOStar Plus microplate reader (BMG Labtech) at 37 °C. Luminescence was measured at 450 ± 40 nm (NLuc intensity), 650 ± 50 nm (HaloTag BRET intensity), and 640 ± 50 nm (Cyofp1 BRET intensity).

For the EA screening assay, the assay media consisted of clear DMEM (GenDEPOT, CM004-310) supplemented with 0.5% FBS, 1% of 100 U/ml penicillin, and 100 μg/ml streptomycin. Stable cells were cultured in 75T flasks (SPL, 70075) and harvested prior to the assay. The cells were resuspended in assay media, and 50,000 cells were added to a white solid-bottom 96-well half-area plate (Greiner Bio-One, 675083) and incubated overnight. The endurazine substrate (final dilution 1:100) was injected into the wells 2 h before the measurement. After 2 h, luminescence was measured to obtained data before treatment, and then the wells were treated with E2 and EAs at 1 μM final concentration. Plates were measured at 6, 12, and 24 h after treatment. Luminescence signals were read in a CLARIOStar Plus microplate reader at 37 °C. The filter set for measuring luminescence was 460 ± 40 nm (NLuc intensity) and 615 ± 20 nm (BRET intensity). The sDRCs and logEC values were generated by the GraphPad9 built-in equation: log(agonist) versus response-variable slope. EC10_nBR_, EC50_nBR_, EC90_nBR_, and EA_nBR_ were calculated using GraphPad9. The BRET ratio and nBRs were calculated using the following formulas:

$$\text{BRET ratio}\;\left(\text{CyOFP}1\right)=\left(\frac{\text{BRET}\;\mathrm{Em}.}{\text{NLuc}\;\mathrm{Em}.}\right)$$ (1)

$$\begin{array}{c} \text{BRET ratio}\;\left(\text{Halotag}\right)=\\ \left(\frac{\text{BRET}\;\mathrm{Em}._{\;\text{HaloTag}\;\left(+\right)}}{\text{NLuc}\;\mathrm{Em}._{\;\text{HaloTag}\;\left(+\right)}}-\frac{\text{BRET}\;\mathrm{Em}._{\;\text{HaloTag}\;\left(-\right)}}{\text{NLuc}\;\mathrm{Em}._{\;\text{HaloTag}\;\left(-\right)}}\right) \end{array}$$ (2)

$$\text{Normalized BRET ratio}=\frac{{\text{BRET ratio}}_{\text{experimental gruop}}}{\text{mean of}\;{\text{BRET ratio}}_{\text{control gruop}}}$$ (3)

Em. represents the emission intensity for each filter. HaloTag(−) and HaloTag(+) represent the HaloTag 618 ligand nontreatment and treatment groups, respectively.

### Construction of stable cell lines

Lenti-X 293T cells (Takara, 632180) were used for lentiviral production to construct a stable cell line using a third-generation lentiviral system. HEK-293 cells in 70-mm culture dish at 90% confluency were transiently transfected with 3.2 μg of the transfer vector, 2.3 μg of pMDLg/pRRE (Addgene #12251), 1.1 μg of pRSV-Rev (Addgene #12253), and 1.5 μg of pMD2.G (Addgene #12259). Lentiviruses were harvested 48 to 60 h after transfection and filtered through a 0.45-μm surfactant-free cellulose acetate (SFCA) membrane (Corning, 431220). The viral supernatants were immediately added to Lenti-X 293T cells using 5 μg/ml polybrene (Santa Cruz Biotechnology, sc-134220A) for transduction. After 3 days, the transduced cells were selected with 1 μg/ml puromycin (Cayman, 13884). The α/α ERDDB stable cell line (ERDDB S.C) was developed by the Ministry of Food and Drug Safety of the Republic of Korea.

### Image acquisition and microscopy

Before imaging, stable cells were cultured in cover glass-bottomed dishes (SPL, 200350). HaloTag 618 ligand and Hoechst stain were added (final dilution, 1:1,000) 8 h after imaging. Cells were imaged using a Leica Dmi8 fluorescence microscope equipped with an LED8 light source, an HC PL APO 40×/1.30 oil immersion objective, DIC module, K5-sCMOS camera, and a CO_2_/37 °C incubation system. For HaloTag 618 imaging, a 642-nm light and CYR71010 filter cube (Leica, 11525416) and a 642-nm filter wheel (Leica, EFW-LED8) were used. For Hoechst imaging, 390-nm light, a DFT51010 filter cube (Leica, 11525418), and a 440-nm filter wheel were used. Merged images were generated using Las X software (Leica, Germany).

### Luciferase reporter and cell viability assay

Luciferase reporter assay was conducted as previously described [18]. Briefly, MCF-7 cells were seeded at 15,000 cells/well in 96-well plates in phenol red-free medium supplemented with 5% charcoal-stripped FBS (csFBS) (Gibco, A3382101). After 24 h, the cells were transfected with 3× ERE TATA Luc (#11354; Addgene) using Lipofectamine 3000 (L3000001; Invitrogen). The transfected cells were incubated for 5 h and then treated with EAs at 1 μM final concentration. After 24 h of incubation, 50 μl of Promega Bright-Glo luciferase substrate dissolved in lysis buffer (Promega, E2610) was added to the cells. For the cell viability assay, MCF-7 cells were seeded at 15,000 cells/well in 96-well plates in a phenol red-free medium supplemented with 5% csFBS and 10 nM E2. After 24 h, the cells were treated with 1 μM EAs. Cell viability was determined using the CellTiter-Glo Luminescent Viability Assay kit (Promega, G7570) after 48 h of incubation. The luminescence was measured using a CLARIOStar Plus microplate reader.

### Statistical analysis

All results are expressed as the mean ± standard deviation (SD) or standard error of the mean (SEM). Statistical significance was assessed using one- or two-way analysis of variance (ANOVA). Statistical significance was set at *P < 0.05, **P < 0.01, ***P < 0.001, and ****P < 0.0001. The fold change was calculated in the same manner as for the nBR.

## Results

### Development and optimization of ERDDBs

**Fig. 1.**
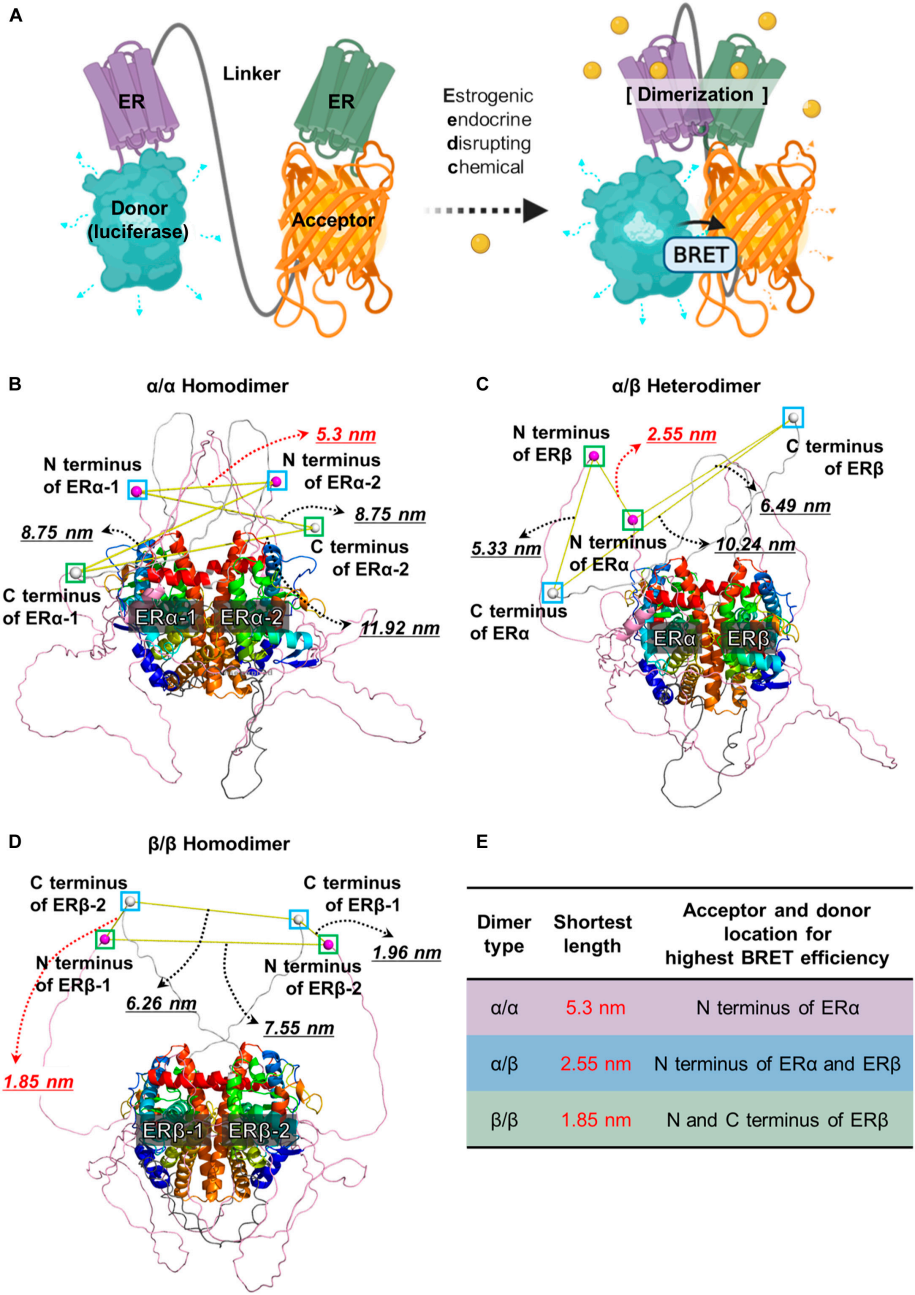
Development and optimization of ERDDBs. (A) Schematic drawing for the activation mechanism of ERDDB. Predicted structures of the (B) ERα/α homodimer, (C) ERα/β heterodimer, and (D) ERβ/β homodimer. The N and C termini are indicated by purple and white spheres, respectively. Yellow lines indicate the distance between ER terminals. The distance is shown next to the dotted arrows. Among the distances, the shortest length is colored by red dotted arrows and red letters. From the C terminus to the N terminus, the ER monomer is colored with a rainbow gradation. The image was generated by PyMOL. (E) Summary of acceptor and donor locations for the highest BRET efficiency according to dimer types. (A) Schematic drawing produced by BioRender (http://biorender.com/).

To enable the real-time detection of ER dimerization within live cells, we designed a novel ERDDB employing the full-length human ER. ERDDB was constructed using a luciferase-based donor and acceptor, each strategically tagged at either the N or C terminus of the ER monomer and connected by a central linker. ERDDB was engineered in such a way that when E2 or an eEDC binds to the ER and induces dimer formation, the proximity between the donor and acceptor diminishes. This leads to the BRET phenomenon, resulting in an increase in the BRET ratio (Fig. 1A). It is noteworthy that the BRET phenomenon necessitates donor–acceptor distances of less than 10 nm, with shorter distances yielding more pronounced effects [19].

To enhance the efficiency of our ERDDB, we conducted a computational structural analysis to identify the most favorable positions for the N or C termini of the donor and acceptor during ER dimerization. AlphaFold, a renowned tool for predicting protein structures from DNA sequences, was employed for this purpose [20,21]. To predict the structure of the full-length ER dimer, we utilized the full-length structures of the ERα and ERβ monomers as predicted by AlphaFold. These structures were then overlaid onto the previously known ER LBD dimer structure obtained from the Protein database to reconstruct the α/α (Fig. 1B), α/β (Fig. 1C), and β/β (Fig. 1D) dimer configurations. Subsequently, the distance between the termini of the ER monomers was measured and the shortest distance was selected. Our results indicated that the highest BRET efficiency was anticipated under the following conditions: for α/α homodimers, the donor and acceptor should be positioned at the N terminus of ERα; for α/β heterodimers, at the N terminus of ERα and the N terminus of ERβ; and for β/β homodimers, at the N and C termini of ERβ (Fig. 1E). Based on the outcomes presented in Fig. 1E, we determined the sequence of ERDDBs to be used in our study.

### Optimization of the acceptor and linker: HaloTag and P2A peptide exhibit superior sensitivity for ERDDB

After establishing the ERDDB sequence, we focused on optimizing the individual components of the sensor, namely, the donor, acceptor, and linker. In this context, we selected Nano Luciferase (NLuc), which is recognized for its exceptional performance [22], as the donor because of its compact size and high brightness. Therefore, our optimization efforts concentrated solely on the acceptor and linker.

**Fig. 2.**
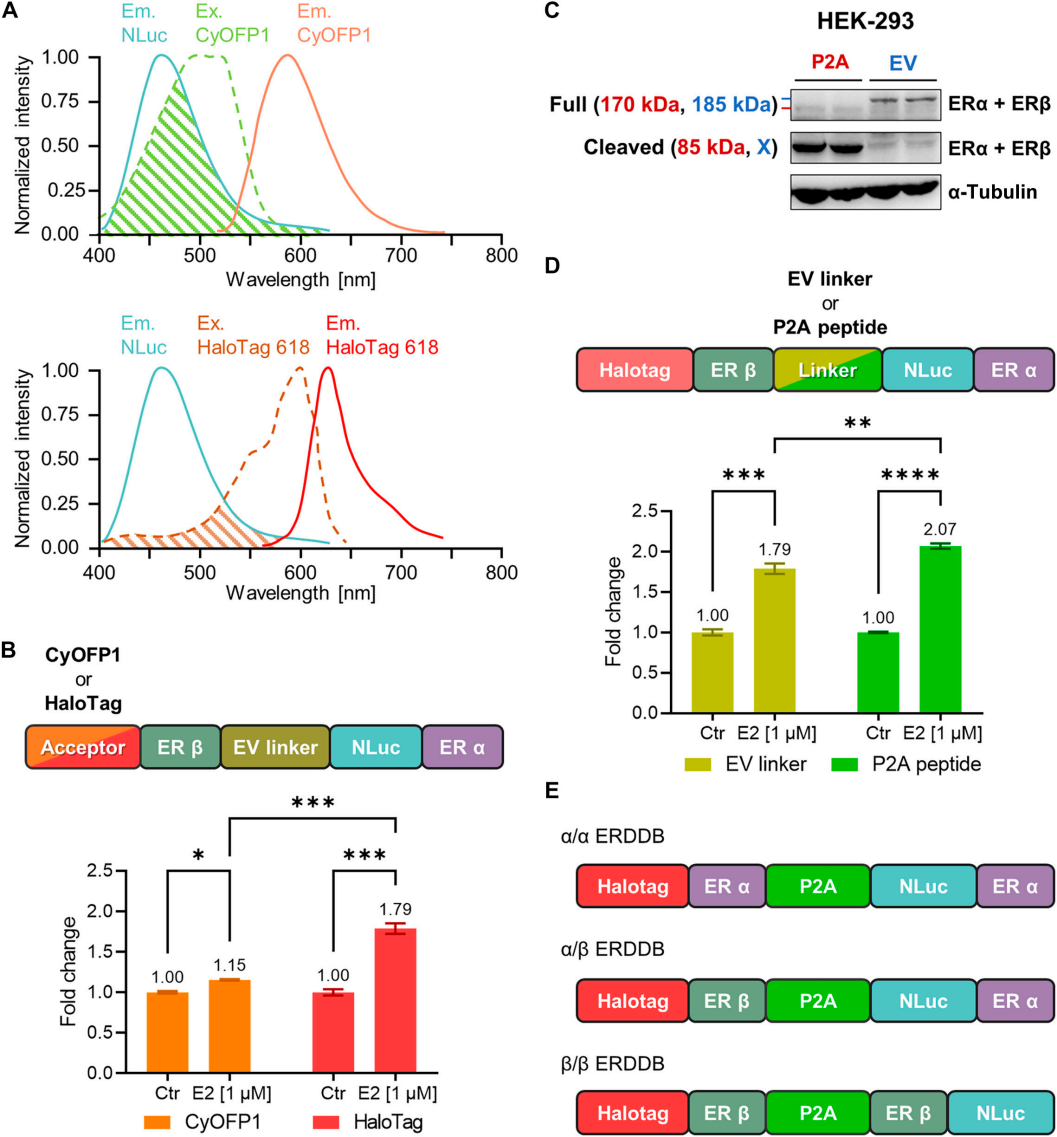
Optimization of the acceptor and linker: HaloTag and P2A peptide exhibit superior sensitivity for ERDDB. (A) Upper: Normalized CyOFP1 excitation (light green dashed line) and emission (orange solid line) spectra. Lower: Normalized HaloTag 618 excitation (brown dashed line) and emission (red solid line) spectra. Both spectra overlaid with the normalized luminescence emission spectrum of NLuc (cyan solid line). The hatched area indicates the overlap area between the emission spectrum of NLuc and the excitation spectrum of CyOFP1 (light green hatched area) or HaloTag 618 (brown hatched area), respectively. Ex. and Em. stand for excitation spectrum and emission spectrum, respectively. (B) Upper: Schematic drawing of used prototype biosensors for comparing acceptor candidates. Lower: Fold change of the CyOFP1 (orange bar) and HaloTag (red bar) acceptor groups (*n* = 2, *P < 0.05, ***P < 0.001, two-way ANOVA test). (C) Immunoblots of the ERα/β biosensor EV linker and P2A peptide forms. Blue and red letters indicate the EV linker and P2A peptide forms, respectively. (D) Upper: Schematic drawing of the used prototype biosensors for comparing linker candidates. Lower: Fold change of the EV (dark yellow bar) and P2A peptide (green bar) linker groups (*n* = 2, **P < 0.01, ***P < 0.001, ****P < 0.0001, two-way ANOVA). (E) Schematic drawing of the final biosensor sequences of the (upper) ERα/α, (middle) ERα/β, and (lower) ERβ/β ERDDBs, respectively. The control group was treated with DMSO, and the experimental group was treated with 1 μM E2. Measurements were conducted 24 h after treatment. Data are represented as the mean ± SEM. The number above the bar represents the mean value.

A variety of acceptor options have been developed for BRET assays, including fluorescent proteins, quantum dots, and more [15,23–26]. From this array of choices, we narrowed our selection to CyOFP1, a fluorescent protein, and HaloTag 618, a fluorescent dye. To validate their suitability for BRET, we confirmed that the excitation spectra of CyOFP1 and HaloTag 618 effectively overlapped with the emission spectra of NLuc. Furthermore, the donor–acceptor distance was sufficient to support the BRET assays. Consequently, CyOFP1 and HaloTag 618 were selected as potential acceptors (Fig. 2A).

CyOFP1 is an orange-red fluorescent protein excited by cyan light. Among the various fluorescent proteins, CyOFP1 stands out for its considerable separation between excitation and emission wavelengths, rendering it an excellent choice for the BRET assay [27]. HaloTag 618 is a member of the HaloTag ligand family, featuring azetidine rings that bind to the HaloTag fusion protein, forming a robust HaloTag complex. This complex remains relatively stable even under challenging conditions, such as acidic microenvironments [28,29]. For these reasons, the NLuc–HaloTag BRET pair has been recognized for its high reliability in studying protein–protein interactions [24].

To conduct a precise comparison of efficiency between CyOFP1 and HaloTag, we constructed prototype ERDDBs by tagging NLuc and HaloTag or CyOFP1 to the N termini of ERα and ERβ monomers, respectively, with an EV linker connecting them (Fig. 2B, upper). Subsequently, we transfected these biosensors into HEK-293T cells, which exhibit low endogenous ER expression, to avoid interference with biosensor function. Comparing the experimental group treated with 1 μM E2 to the control group, we calculated a fold change, revealing a 15% and 79% change for CyOFP1 and HaloTag, respectively. Importantly, this represents an approximately 5.26-fold higher change rate for the HaloTag than for the CyOFP1 acceptor form (Fig. 2B, lower).

The EV linker, characterized by its extended and flexible structure spanning 116 amino acids, has been demonstrated to minimally affect ER binding while ensuring the physical connection of the two ER monomers. This physical connection enhances the intended biosensor response, facilitating its occurrence [30–32]. In contrast, the P2A peptide are self-cleaving peptide employed in generating cistronic plasmid constructs, thus ensuring the expression of both ER monomers in a 1:1 ratio within a single construct [31,33]. This approach results in a more precise biosensor expression compared to individually transfecting each ER monomer. Furthermore, the P2A peptide enables biosensors to mimic the physiological behavior of the ER, as the endogenous ER exists in a monomeric form and forms dimers upon binding to E2.

To confirm the functionality of both linkers, immunoblot analysis was performed. The results indicated that the EV linker form displayed a band at the ERα/β EV linker (185 kDa), while the P2A peptide form displayed bands at NLuc-ERα (85 kDa) and CyOFP1-ERβ (85 kDa). This confirmed that biosensor cleavage occurred correctly in the P2A linker form, in contrast to the EV linker form (Fig. 2C). To determine which linker would yield higher biosensor efficiency, we created two forms of prototype biosensors, one with an EV linker and the other with a P2A peptide linker (Fig. 2D, upper). Employing the same experimental methodology, we calculated the fold change, revealing 79% and 107% changes for the EV linker and P2A peptide, respectively. Notably, this represents an approximately 1.35-fold higher rate of change for the P2A peptide linker than for the EV linker (Fig. 2D, lower). Based on these results, we selected HaloTag as the acceptor and the P2A peptide as the linker. These components were applied to the other ERDDB types, including α/α and β/β configurations, for the construction of optimized ERDDBs.

### Validation of ERDDB responsiveness to ER dimerization-inducing agents

**Fig. 3.**
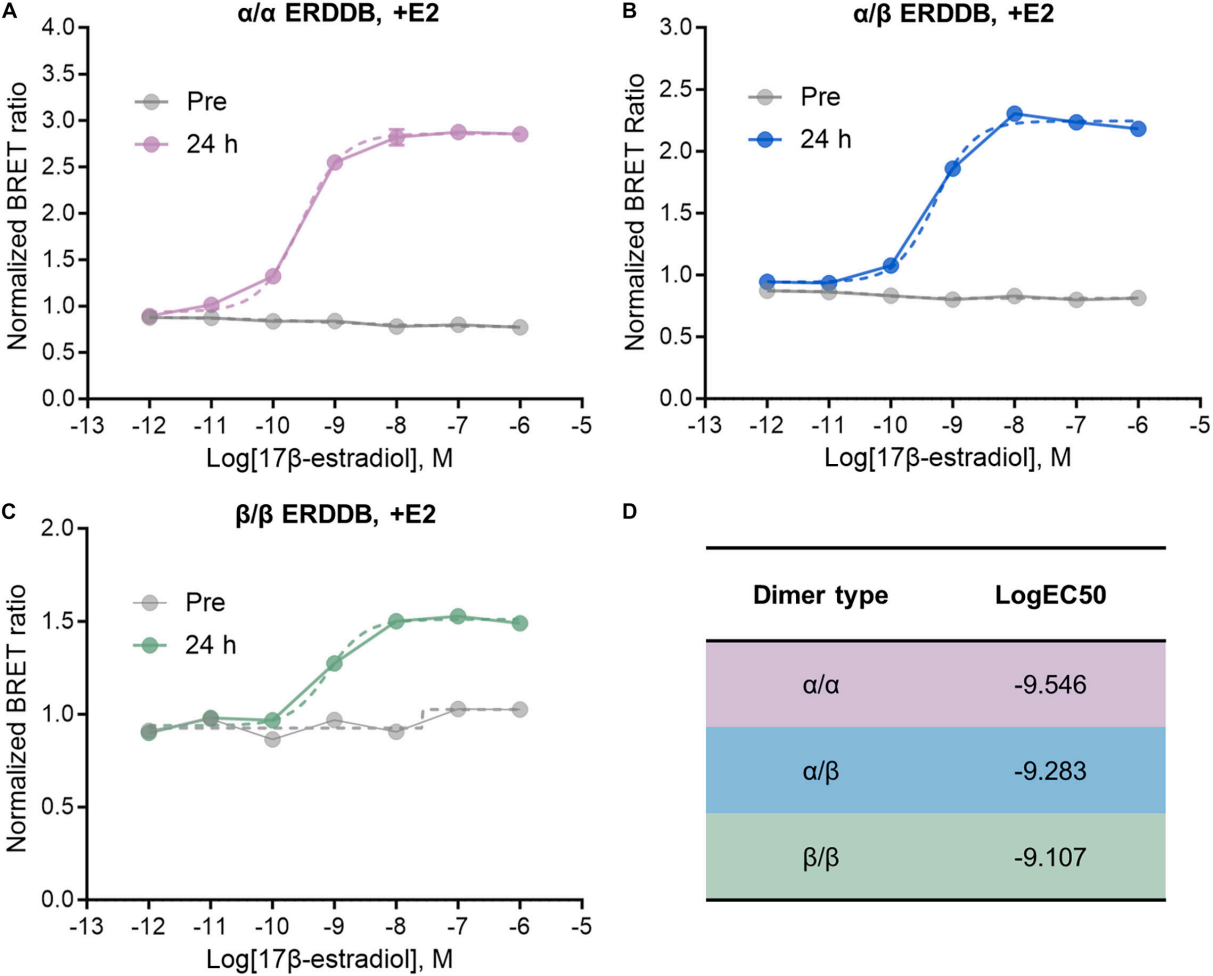
Validation of ERDDB responsiveness to E2. The sDRC of the (A) α/α, (B) α/β, and (C) β/β ERDDBs for serially diluted E2 treatment. Pre and 24 h mean before and 24 h after E2 treatment. Treatment with a 10-fold serial dilution of E2, ranging from 1 μM to 1 pM, or DMSO was conducted. The dashed line indicates the dose–response curve. (D) Summary of the differences in EC50 values for each ERDDB. Fold changes in the EC values were calculated by converting the difference in the logEC value to the exponent value. The sDRC and LogEC50 value were generated by the following GraphPad9 built-in equation: log(agonist) versus response-variable slope. All error bars represent the SEM. If the error bar was smaller than the symbol size, it was not displayed on the graph.

To assess the accuracy of the optimized ERDDBs in faithfully representing biological ER dimerization responses, we constructed sigmoidal dose–response curves (sDRCs) using E2 and expressed them with normalized BRET ratios (nBRs). We observed notable differences in the sDRC before and after 24 h of E2 treatment for the α/α (Fig. 3A), α/β (Fig. 3B), and β/β (Fig. 3C) ERDDBs. The logged half-maximal effective concentration (LogEC50) values for the α/α, α/β, and β/β ERDDBs were calculated as −9.546, −9.283, and −9.107, respectively (Fig. 3D). These findings substantiate the ability of ERDDBs to accurately reflect ER dimerization in response to E2.

To ascertain whether ERDDBs selectively respond to substances that induce dimerization rather than to all ligands, we generated sDRCs using various ER-related drugs. Fulvestrant, a selective ER degrader, induces ER degradation in ER-positive cells by binding to the receptor [34–36]. In ER-transfected cells, it exhibits the opposite effect, protecting ER from degradation and promoting the formation of insoluble ER aggregates, consequently increasing dimerization [37,38]. ERDDBs demonstrated a dose-dependent increase in the BRET ratio in response to fulvestrant (Fig. S1A). 4-Hydroxytamoxifen, a metabolite of the anti-estrogen drug tamoxifen, competitively binds to the ligand-binding domain of ER, leading to ER dimerization [39]. As shown in Fig. S1B, the BRET ratio increased in a dose-dependent manner. Similarly, H3B-5942, a covalent ERα antagonist derived from the structure of 4-hydroxytamoxifen [40], elicited a dose-dependent sigmoidal response in all three ERDDB types, with the highest fold change observed in ERα/α (Fig. S1C).

In the case of ER agonists, synthetic estrogens, such as diethylstilbestrol and dienestrol, displayed dose-dependent sigmoidal curves (Fig. S1D and E). Remarkably, α-estradiol, a weak endogenous steroidal estrogen, yielded a relatively lower BRET ratio and a higher EC50 compared to E2 (Fig. S1F). In contrast, angiotensin, categorized as a phytoestrogen, resulted in a marginal increase in the BRET ratio only at the highest concentration of 1 μM (Fig. S1G). This finding aligns with a previous study indicating that 1 μM angiotensin is insufficient to induce ER dimerization and transactivation [41]. Lastly, corticosterone, employed as an agonist negative control in the OECD TG 455 [14], demonstrated no impact on the BRET ratio across all treatment concentrations (Fig. S1H). These results confirmed that ERDDBs specifically respond to ER dimerization-inducing drugs.

### Construction and validation of ERDDB stable cell lines for screening

**Fig. 4.**
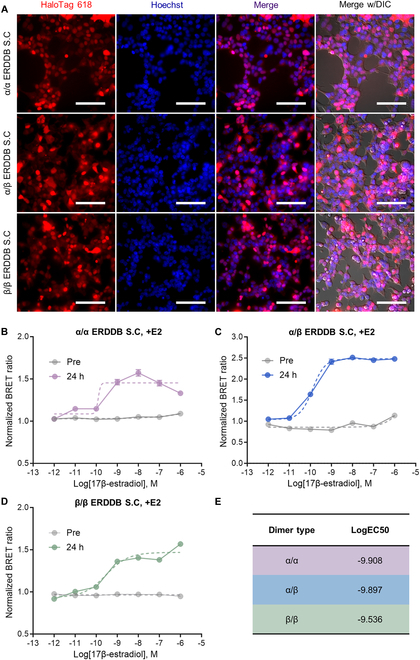
Construction and validation of ERDDB S.Cs for screening. (A) Fluorescence images of all dimer types of ERDDB S.Cs. ERDDB-expressing cells (HaloTag 618, red), entire cells (Hoechst, blue), merged image, and merged image with DIC. All images were acquired using a 40× objective lens. The white scale bar represents 100 μm. ERDDB S.Cs showing the sDRC by E2 treatment in (B) ERα/α, (C) ERα/β, and (D) ERβ/β dimers. Pre means before treatment with E2, and 24 h means 24 h after E2 treatment. Treatment was conducted with a 10-fold serial dilution of E2, ranging from 1 μM to 1 pM, or DMSO. (E) Summary of the differences in EC50 values for each ERDDB S.C. Fold changes in the EC values were calculated by converting the difference in the logEC value to the exponent value. The sDRCs and LogEC50 values were generated by the following GraphPad9 built-in equation: log(agonist) versus response-variable slope. All error bars represent the SEM. If the error bar was shorter than the symbol size, it was not displayed in the graph. The dashed lines represent the sDRCs. S.C stands for stable cell line.

Cell-based screening assays offer a biologically relevant and efficient means of assessing a wide range of potential eEDC candidates that induce ER dimerization. Stable cell lines are commonly employed in screening assays because of several advantages. First, stable cell lines maintain relatively stable protein expression, reducing signal variability and ensuring data reliability in repeated experiments. Second, they eliminate the need for transient transfection, thereby simplifying large-scale screening processes. Third, the use of stable cell lines helps to minimize human error, resulting in consistent outcomes that can be replicated by multiple institutions and researchers. To establish the applicability of ERDDBs for screening, we generated stable cell lines using the third-generation lentivirus system, followed by puromycin selection. To confirm the successful implementation of puromycin selection, the ERDDB S.C was stained with the HaloTag 618 ligand to label ERDDB-expressing cells, whereas Hoechst was used to stain all cells. Using fluorescence imaging, we observed that ERDDBs were stably expressed in both the nucleus and cytoplasm of most cells (Fig. 4A).

To ascertain the stable and functional expression of ERDDBs within the cells, we constructed sDRCs using the same method as in previous experiments, employing E2 in the α/α (Fig. 4B), α/β (Fig. 4C), and β/β (Fig. 4D) ERDDB S.Cs. LogEC50 values were calculated as −9.908, −9.897, and −9.536, respectively (Fig. 4E). Notably, all ERDDB S.Cs displayed lower LogEC50 values than transiently transfected ERDDBs (Figs. 3D and 4E). These findings confirm the successful construction of ERDDB S.Cs and demonstrate their increased sensitivity in detecting E2 and eEDCs.

### ERDDB can discriminate estrogen analogs inducing ER dimerization using E2 standard graph-based criteria for each dimer type

To establish the criteria for identifying ER dimerization inducers, we used the E2 sDRC as an E2 standard graph. The use of an E2 standard graph for criterion determination offers several advantages. First, it compensates for the experimental variability arising from different cellular conditions because the standard graph reflects these variables and can be employed for data compensation. Second, it permits cross-validation based on the sDRC shape and EC50 values, thus ensuring the reliability of the results in repeated experiments. Finally, specific criteria are essential for each dimer type when comparing the screening results for the three types of dimers because the dynamic range of the nBR varies depending on the dimer type.

**Fig. 5.**
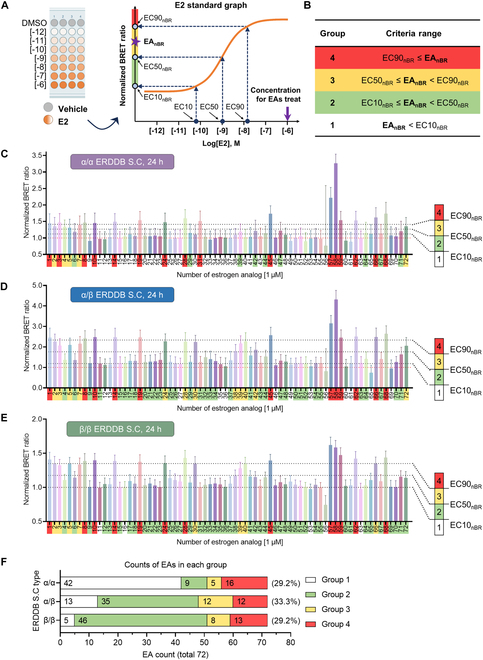
ERDDB discriminates EAs inducing ER dimerization using E2 standard graph-based criteria for each dimer type. (A) Schematic drawing of the calculating criteria. The E2 standard graph was generated using a 10-fold serial dilution of E2 as treatment (orange solid line). EC10, EC50, and EC90 values were converted to nBRs using the E2 standard graph. First, the EC values were computed using the E2 standard graph and the location on the *x* axis was found (navy blue filled circle). Second, the EC_nBR_ values (sky blue filled circle) were obtained by following the dashed arrows (navy blue dashed arrow), which compute the *y* value corresponding to the *x*-axis value on the E2 standard graph. Last, the EC_nBR_ and EA_nBR_ (purple star) values were compared to determine to which group the EAs belong. All EAs were used at 1 μM for treatment. (B) To classify EAs, the group was set according to criteria ranges. Criteria ranges were set using the EC_nBR_ and EA_nBR_ values. Groups 1, 2, 3, and 4 were colored with white, green, yellow, and red, respectively. Results of EA screening using the (C) α/α, (D) α/β, and (E) β/β ERDDB S.Cs 24 h after EA treatment. See also Table S1 for estrogen analog information. The numbers in the vertical bar represent the group number with their colors. Black dotted lines indicate EC90_nBR_, EC50_nBR_, and EC10_nBR_ from top to bottom, respectively. The red dotted line indicates 1, which is the nBR of the vesicle control group. The background color of the EA number was determined according to the group color in (B). All results are expressed as the mean ± SD. SC stands for stable cell line. nBR stands for normalized BRER ratio. (F) The numbers in the bar graph represent the EA counts in each group. Percentages next to the bar graph represent the proportion of EAs that respond above the EC50_nBR_ in each dimer type.

For further validation of ERDDB S.Cs, we used 72 test substances, hereafter referred to as estrogen analogs (EAs), recommended by the National Institutes of Health for validation of in vitro EDC detection methods [42]. For the screening process, we aimed to use a high concentration because we prioritized minimizing false negatives, which is crucial as false positives can be further validated using the additional screenings with the ER binding (TG 493) [13] and ER transactivation (TG 455) [14] assays. In this regard, we selected a concentration of 1 μM, a level significantly surpassing the EC50 of E2 by over a thousandfold. The results were expressed as the nBR of each EA (EA_nBR_), rather than EC50 values, so a conversion process was necessary to compare the EC50 value of the E2 standard graph with that of EA_nBR_. This conversion was accomplished by calculating the nBR corresponding to the EC50 value (EC50_nBR_), which involved determining the *y*-axis value corresponding to the EC value on the *x* axis of the standard graph. Additionally, for a more detailed classification of EAs based on their responsiveness, we computed the EC10_nBR_ and EC90_nBR_ values using the same method to denote low and high responsiveness, respectively (Fig. 5A). These EC_nBR_ values facilitated a more granular categorization of EAs. The EC50_nBR_ values for each ERDDB dimer type were represented as α/α EC50_nBR_, α/β EC50_nBR_, and β/β EC50_nBR_.

EAs were grouped into four categories based on the EC_nBR_ values, with the criteria ranges defined as follows: group 1, EA_nBR_ < EC10_nBR_; group 2, EC10_nBR_ ≤ EA_nBR_ < EC50_nBR_; group 3, EC50_nBR_ ≤ EA_nBR_ < EC90_nBR_; group 4, EC90_nBR_ ≤ EA_nBR_ (Fig. 5B). The screening results revealed the counts of EAs that exhibited responses equal to or greater than the EC50_nBR_ at 24 h after treatment, with 21, 24, and 21 in the α/α, α/β, and β/β ERDDB S.C, respectively. These accounted for 29.2%, 33.3%, and 29.2% of the total EAs, respectively (Fig. 5C to F). Interestingly, while the calculated percentages were similar, the counts of EAs falling into group 2 increased from the β/β dimer type to the α/α dimer type. These data suggested that EAs elicit varying responses depending on the type of ER dimer.

### The ERDDB screening assay enables subtype-specific classification of EAs via ER dimer type cross-comparison

**Fig. 6.**
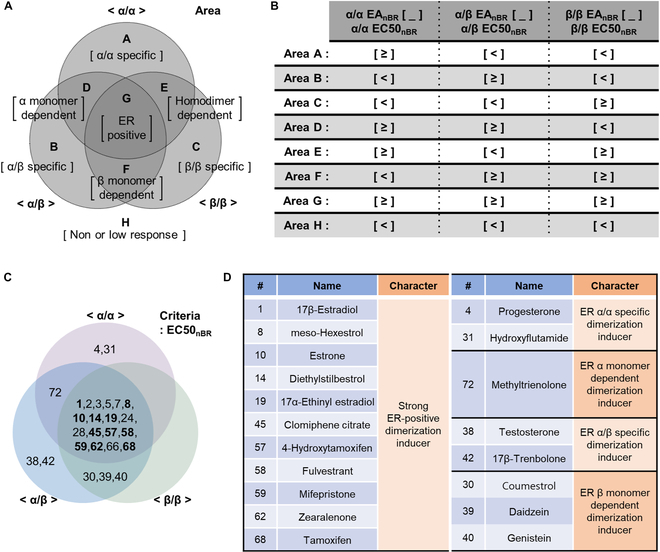
ERDDB screening assay enables subtype-specific classification of EAs via ER dimer type cross-comparison. (A) Area layout of the Venn diagram and its character. Each gray circle represents ER dimers. Each area is bordered with black dashed lines and marked with capital letters. The character of each area is shown in square brackets. (B) The table summarizes the criteria for selecting EAs corresponding to each area in (A). The EAs were categorized based on the combination of EA responses according to every ER dimer type. To read the table, substitute the inequality sign into the square brackets of the formula in the top row (for example, EAs in group G should satisfy three conditions: α/α EA_nBR_ ≥ α/α EC50_nBR_, α/β EA_nBR_ ≥ α/β EC50_nBR_, and β/β EA_nBR_ ≥ β/β EC50_nBR_). (C) Venn diagram illustration of the classified EAs by condition. The EAs are classified according to the layouts in (B). In group G, if EA_nBR_ is higher than EC90_nBR_ in all ER types, the number of EAs is bolded. EAs included in group H, which is composed of nonresponses or low responses, are listed in Table S2. (D) Name and characters of notable EAs. The other EA numbers are listed in Table S1.

Because all ERDDB dimer types share a common foundation, a comparison of EAs classified from ERDDB S.Cs becomes feasible. To facilitate this, we constructed a Venn diagram employing three circles, each representing an ER dimer type, and identified seven distinct areas characterizing the EAs (Fig. 6A). EAs that selectively induce α/α, α/β, or β/β dimers are included in areas A, B, and C, respectively. If dimerization depends on the ERα monomer, these EAs fall into area D, whereas if it relies on the ERβ monomer, they belong to area E. EAs inducing homodimer formation independently of ERα or ERβ are situated in area F. For EAs that induce dimerization across all ER dimer forms, they occupy area G, whereas those with a low or negligible response are found in area H (Fig. 6A).

To identify the EAs corresponding to each area shown in Fig. 6A, we compared the EA_nBR_ and EC50_nBR_ values for each dimer type (Fig. 6B). The table in Fig. 6B shows the criteria for the EAs to be situated within each area in Fig. 6A. The table is structured with three inequality signs for each area, and the formulas for each dimer type are enclosed in square brackets, which serve as placeholders for the inequality signs in the top row. For instance, EAs categorized in area A, according to the characteristics of Fig. 6A, should demonstrate a heightened response in the α/α ERDDB S.C, while displaying lower responses in the α/β and β/β ERDDB S.C. Consequently, EAs in area A must satisfy three criteria simultaneously: α/α EA_nBR_ ≥ α/α EC50_nBR_, α/β EA_nBR_ < α/β EC50_nBR_, and β/β EA_nBR_ < β/β EC50_nBR_. Additionally, we can classify EAs with strong responses based on EC90_nBR_. In particular, the EAs included in area G, those exhibiting responses surpassing EC90_nBR_ in all dimer forms, are indicated in bold (Fig. 6C).

Finally, we successfully classified 26 EAs; the selection of notable EAs is summarized in a table (Fig. 6D). We identified 18 EAs as ER-positive dimerization inducers, with 11 of them displaying particularly robust induction. Furthermore, progesterone and hydroxyflutamide were categorized as ERα/α-specific dimerization inducers, while testosterone and 17β-trenbolone were identified as ERα/β-specific dimerization inducers. Methyltrienolone was classified as an ERα monomer-dependent dimerization inducer, while coumestrol, daidzein, and genistein were identified as ERβ monomer-dependent dimerization inducers (Fig. 6D).

## Discussion

In this study, we developed fine-tuned BRET-based biosensors capable of detecting ER homodimers and heterodimers, enabling the classification of ER dimer subtype-specific EAs. This breakthrough holds promise for advancing ER subtype-specific drug screening with the potential to mitigate the side effects associated with nonspecific interventions.

While previous studies have predominantly concentrated on evaluating ERα dimerization exclusively [43–45], our biosensors offer a comprehensive assessment by encompassing both ER homodimers and heterodimers. The utilization of stable cell lines established using lentiviral technology significantly contributes to the reliability and reproducibility of our findings. This approach eliminates the potential variability introduced by transient transfection and subsequent cell passaging, thus ensuring the robustness of our methodology for future investigations. The incorporation of BRET technology, particularly in tandem with NLuc, markedly increases the sensitivity of the biosensors. This results in an elevated signal-to-noise ratio, facilitating the precise assessment of potential eEDCs, especially in low-concentration scenarios. Our investigation reveals that the fusion of NLuc with HaloTag, in contrast to fusion with CyOFP1, produces a more robust signal. Furthermore, we identified an optimal linker for efficient application in biosensors, further augmenting their overall performance. In addition, the real-time attributes of live-cell experiments enable dynamic and time-dependent observations of EDC responses over extended periods. This capability provides invaluable insights into the temporal dynamics of EDC interactions with the endocrine system, thereby enriching our understanding of their effects on cellular processes. Collectively, these advantages underscore the significant contribution of our biosensor to the advancement of EDC detection in the research domain.

The sDRC demonstrates a plateau phase at high concentrations owing to reaction saturation. Similarly, in the Fig. S2 graph, certain EAs exhibit insignificance when compared to E2. Although further investigation is required, it is hypothesized that these EAs may have reached a plateau. Conversely, a subset of EAs demonstrated exceptional significance compared with E2, displaying remarkably high signals. Notably, 4-hydroxytamoxifen and fulvestrant consistently exhibited pronounced significance across all dimer types. We hypothesized that these drugs may overcome saturation by inducing conformational changes in the ER. Indeed, prior research has established the ability of 4-hydroxytamoxifen and fulvestrant to induce structural changes in the ER [36,46]. This implies that ERDDB holds promise in identifying EAs capable of inducing structural alterations in the ER.

In our extensive assessment of 72 EAs using ERDDBs in conjunction with the ERE-luciferase reporter assay, we observed noteworthy consistency in trends compared to both previous research and the OECD test guidelines. For instance, 4-hydroxytamoxifen, a representative selective ER modulator, is known to bind to the ER with high affinity and induce ER dimerization [47,48], acting as an ER antagonist by blocking the activation function-2 domain of the ER [49]. Our data align with these characteristics, demonstrating the induction of ER dimerization (Fig. 6C), while showing no concurrent activation of the ERE (Fig. S3A). In addition, atrazine, which interferes with the binding of E2 to the ER as per TG 493 [13], exhibited no discernible impact on ER dimerization (Fig. 6C), ERE activation (Fig. S3A), or E2-induced cell proliferation (Fig. S3B) in our study. This consistency with previous research reinforces the notion that while atrazine inhibits the binding of E2 to the ER [50], it does not affect ERE activity and E2-induced MCF-7 cell proliferation [51]. Another example is actinomycin D, a chemotherapy medication, which, according to our data, diminished ERE activity (Fig. S3A) and E2-induced cell proliferation (Fig. S3B) without inducing ER dimerization (Fig. 6C). This observation aligns with previous studies indicating that actinomycin D prevents nuclear processing of ER by intercalating in DNA to inhibit ERE-mediated transactivation [52,53]. The actinomycin D-treated group showed an increase in SD over time in all dimer types, presumably due to cytotoxicity (Fig. S4). These findings not only confirm the accuracy of our methodology but also enhance its credibility as a valuable tool for detecting eEDCs.

The OECD advocates prioritizing the acquisition of reliable mechanistic in vitro data for detecting EDCs instead of resorting to in vivo assays to minimize unnecessary animal usage [54]. Recognizing that in vitro assays may not fully encapsulate the intricacies of a complete organism, it is imperative to develop assays that can detect eEDCs for each of the ER signaling pathways. Notably, the absence of test guidelines for assessing ER dimerization within the OECD framework emphasizes the need for reliable methods in this domain. Using our newly developed ERDDBs, we systematically evaluated 72 EAs by comparing data on ERE-mediated transcriptional activation and subsequent cell viability. Consequently, our study successfully pioneered the development and optimization of ERDDBs, presenting a promising screening tool for eEDCs that is likely to be incorporated into the OECD test guidelines.

## Conclusions

Our study introduces a robust and efficient tool for the rapid, accurate, and sensitive detection of EDCs. We developed a unified ERDDB platform capable of discerning all types of ER dimers and optimized ERDDBs to improve sensitivity. Additionally, we established stable cell lines expressing ERDDBs and conducted screening assays using a diverse array of EAs. Through cross-comparisons of ER dimers, we successfully identified EAs that specifically induce distinct ER subtypes. The implementation of ERDDBs promises more comprehensive assessments of ER signaling dynamics, aligning with OECD test guidelines and significantly contributing to the progression of environmental and human health research.

## Data Availability

Data are available on request from the authors.
