# Supplementary material for: Novel Estrogen Receptor Dimerization BRET-Based Biosensors for Screening Estrogenic Endocrine-Disrupting Chemicals
Source: Biomater Res. 2024 Mar 7;28:0010. doi: 10.34133/bmr.0010 (PMC10923609; doi:10.34133/bmr.0010)
Supplement: Supplementary 1 — Materials and Methods Tables S1 and S2 Figs. S1 to S4 [file bmr.0010.f1.pdf]

Supplemental information

Novel estrogen receptor dimerization BRET-based biosensors for screening estrogenic endocrine-disrupting chemicals

Gyuho Choi[1†], Hyunkoo Kang[1†], Jung-Soo Suh[1], Haksoo Lee[1], Kiseok Han[1], Gaeun Yoo[1], Hyejin Jo[2], Yeong Min Shin[2], Tae-Jin Kim[1,3*] and BuHyun Youn[1,3,4*]

[1]Department of Integrated Biological Science, Pusan National University, Busan, 46241, Republic of Korea

[2]Food Safety Risk Assessment Division, National Institute of Food and Drug Safety Evaluation, Ministry of Food and Drug Safety, Cheongju, 28159, Republic of Korea

[3]Department of Biological Sciences, Pusan National University, Busan, 46241, Republic of Korea

[4]Nuclear Science Research Institute, Pusan National University, Busan 46241, Republic of Korea

[†]These authors contributed equally to this work.

[*]Correspondence:

Tae-Jin Kim (tjkim77@pusan.ac.kr),

BuHyun Youn (bhyoun72@pusan.ac.kr)

Material and methods

Table S1-S2

Figure S1-S3

Full list of EAs for screening assay

#	Name	#	Name	#	Name
1	17β-Estradiol	25	Procymidone	49	Flavone
2	5α-Dihydrotestosterone	26	Linuron	50	Fluoranthene
3	Bisphenol A	27	Kaempferol	51	Propylthiouracil
4	Progesterone	28	Bisphenol B	52	Sodium azide
5	4-Cumylphenol	29	p,p'-Methoxychlor	53	L-Thyroxine
6	Di-n-butyl phthalate	30	Coumestrol	54	Fenarimol
7	4-tert-Octylphenol	31	Hydroxyflutamide	55	Nilutamide
8	meso-Hexestrol	32	Cyproterone acetate	56	Actinomycin D
9	Ethyl paraben	33	Ketoconazole	57	4-Hydroxytamoxifen
10	Estrone	34	Apigenin	58	Fulvestrant
11	Medroxyprogesterone acetate	35	Dexamethasone	59	Mifepristone
12	Corticosterone	36	Phenolphthalin	60	Pimozide
13	4-Androstenedione	37	Cycloheximide	61	12-O-Tetradecanoylphorbol-13-acetate
14	Diethylstilbestrol	38	Testosterone	62	Zearalenone
15	Flutamide	39	Daidzein	63	Anastrazole
16	Haloperidol	40	Genistein	64	Dibenzo[a,h]anthracene
17	2-sec-Butylphenol	41	Diethylhexyl phthalate	65	Apomorphine
18	Vinclozolin	42	17β-Trenbolone	66	Norethynodrel
19	17α-Ethinyl estradiol	43	Bicalutamide	67	p-n-Nonylphenol
20	Reserpine	44	Methyl testosterone	68	Tamoxifen
21	Spironolactone	45	Clomiphene citrate	69	Morin
22	Butylbenzyl phthalate	46	p,p'-DDE	70	Fadrozole
23	Atrazine	47	o,p'-DDT	71	Kepone (Chlordecone)
24	17α-Estradiol	48	Finasteride	72	Methyltrienolone

Table S1. Full list of the EAs used in the screening assay

The assigned number and name of the EAs used for EA screening are shown.

List of Non or low response EAs

#	Name	#	Name	#	Name
6	Di-n-butyl phthalate	29	p,p’-Methoxychlor	52	Sodium azide
9	Ethyl paraben	32	Cyproterone acetate	53	L-Thyroxine
11	Medroxyprogesterone acetate	33	Ketoconazole	54	Fenarimol
12	Corticosterone	34	Apigenin	55	Nilutamide
13	4-Androstenedione	35	Dexamethasone	56	Actinomycin D
15	Flutamide	36	Phenolphthalin	60	Pimozide
16	Haloperidol	37	Cycloheximide	61	12-O-Tetradecanoylphorbol-13-acetate
17	2-sec-Butylphenol	41	Diethylhexyl phthalate	63	Anastrazole
18	Vinclozolin	43	Bicalutamide	64	Dibenzo[a,h]anthracene
20	Reserpine	44	Methyl testosterone	65	Apomorphine
21	Spironolactone	46	p,p’-DDE	67	p-n-Nonylphenol
22	Butylbenzyl phthalate	47	o,p’-DDT	69	Morin
23	Atrazine	48	Finasteride	70	Fadrozole
25	Procymidone	49	Flavone	71	Kepone (Chlordecone)
26	Linuron	50	Fluoranthene		
27	Kaempferol	51	Propylthiouracil		

Table S2. List of non- or low-response EAs

The assigned number and name of non- or low-response EAs. 46 EAs were sorted. These EAs showed lower EA_{nBR} than $EC50_{nBR}$ in every dimer type. These are the EAs included in area H of Figure 6A.

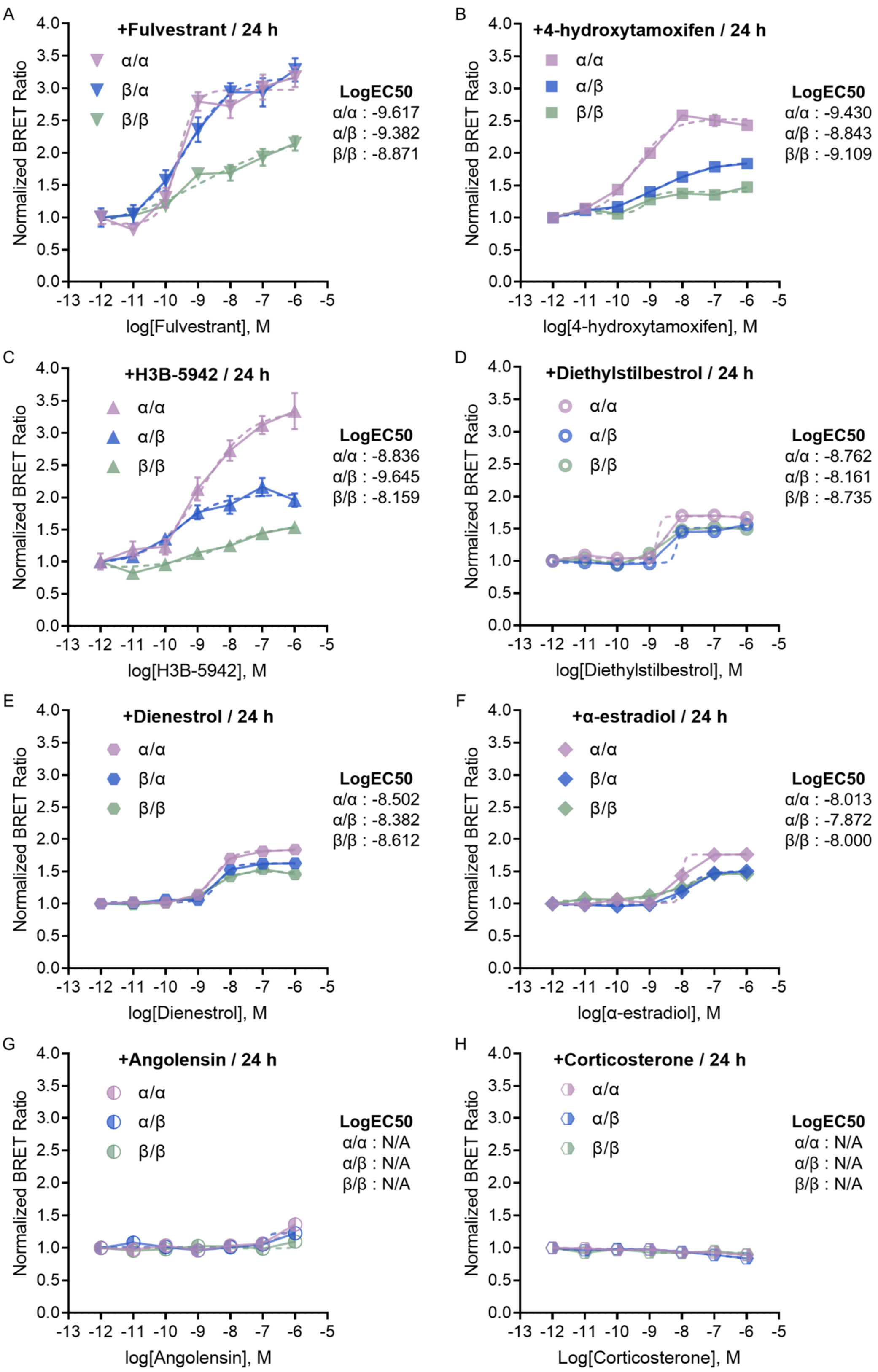

Figure S1. Validation of ERDDB responsiveness to ER dimerization-inducing agents

The sDRCs and LogEC50 values of (A) fulvestrant, (B) 4-hydroxytamoxifen, (C) H3B-5942, (D) diethylstilbestrol, (E) dienestrol, (F) α-estradiol, (G) angiotensin, and (H) corticosterone in

each α/α, α/β, and β/β ERDDB at 24 h post-treatment. Treatment was performed with ten-fold serial dilutions of each drug, ranging from 1 μM to 1 pM, or DMSO. The sDRCs and LogEC50 values were generated by the following GraphPad9 built-in equation: log(agonist) vs. response-variable slope. All error bars represent the SEM. If the error bar was smaller than the symbol size, it was not displayed on the graph. If the confidence interval of the LogEC50 could not be determined, the data were marked as Not Available (N/A). The dash line represents the sDRC.

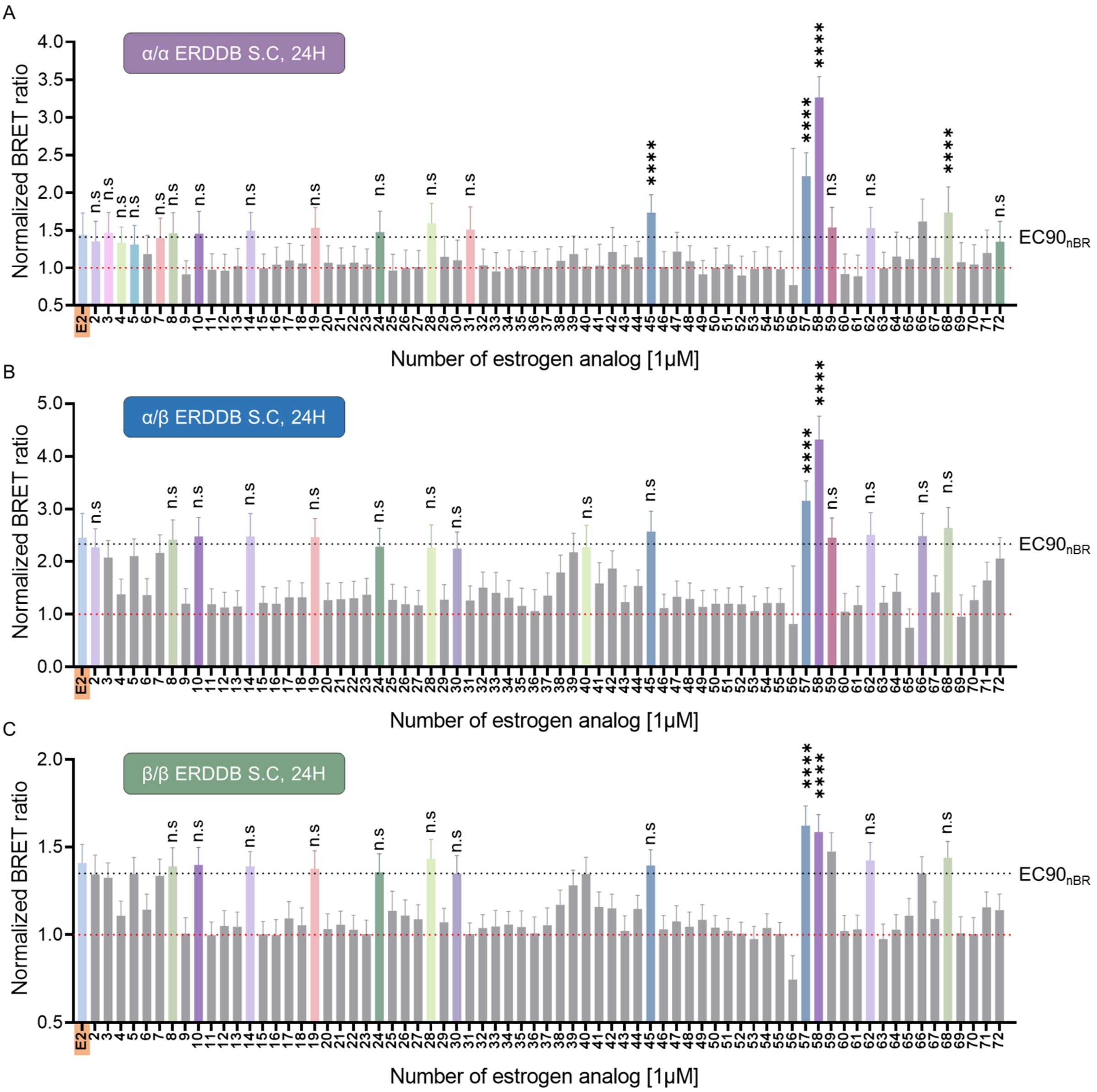

Figure S2. Some EAs exhibit unusually high BRET ratio values above the plateau BRET ratio induced by E2

Non or very highly significant EAs compared to E2 among the highly responsive EAs for the (A) α/α (B) α/β, and (C) β/β ERDDB S.Cs (n.s: not significant, ****: $p < 0.0001$, Brown-Forsythe and Welch One-way ANOVA test). Other EAs showed other significances, such as ***, **, and *, but only n.s and **** are presented in this graph. Each EA was statistically compared with E2 (orange background). E2 was indicated as ‘1’ in the number of EAs in previous data. All results are expressed as the mean ± SD. See also Table S1 for estrogen analog

information. The black dotted lines indicate the $EC90_{nBR}$. The red dotted line indicates 1, which represents the nBR of the vesicle control group. Figures S2A-S2C were constructed using graph data from Figures 5C-5E, respectively.

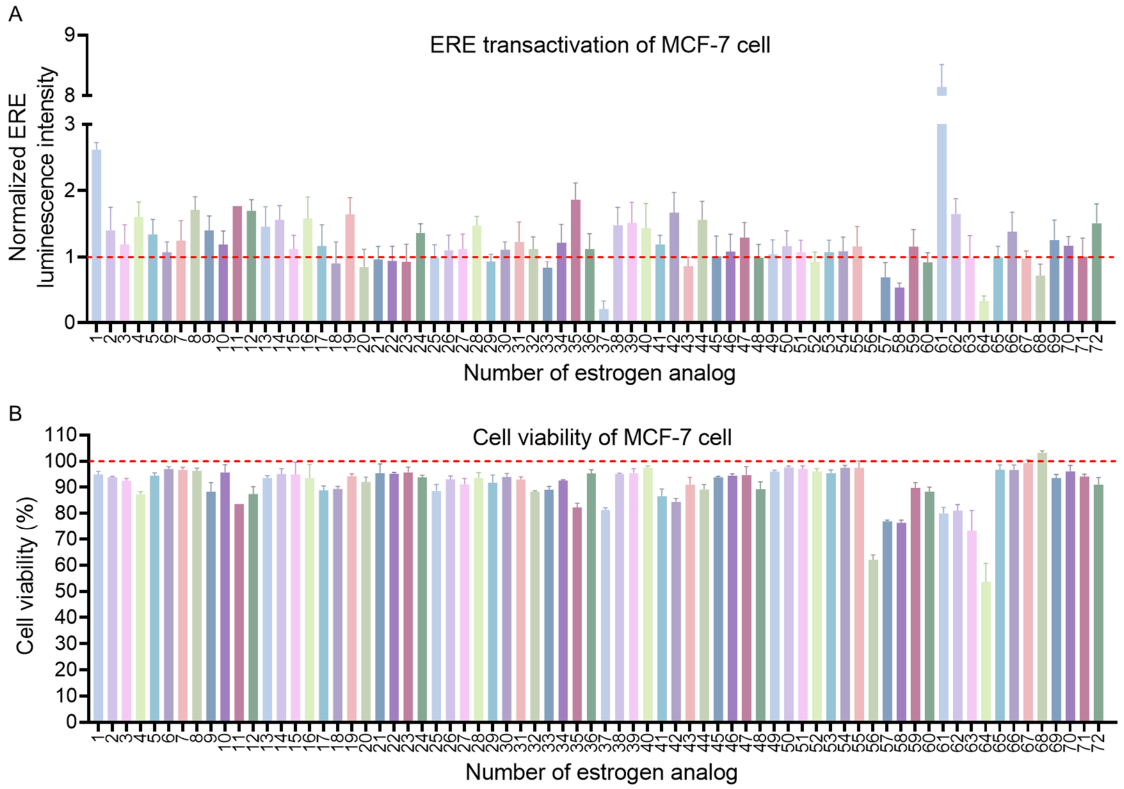

Figure S3. Effect of EAs on ERE transactivation and E2-induced cell proliferation in ERα-positive cells

(A) Luciferase activity following EA treatment upon transfection of a 3X ERE TATA luciferase plasmid in MCF-7 cells. The data were measured 24 h after treatment with 1 μM of 72 different EAs. (B) The cell viability of MCF-7 cells was examined after incubation with 10 nM E2 following EA treatment. The data were measured 48 h after treatment with 1 μM of 72 different EAs. See also Table S1 for estrogen analog information. The red dashed line in each graph represents the value of the vesicle control group.

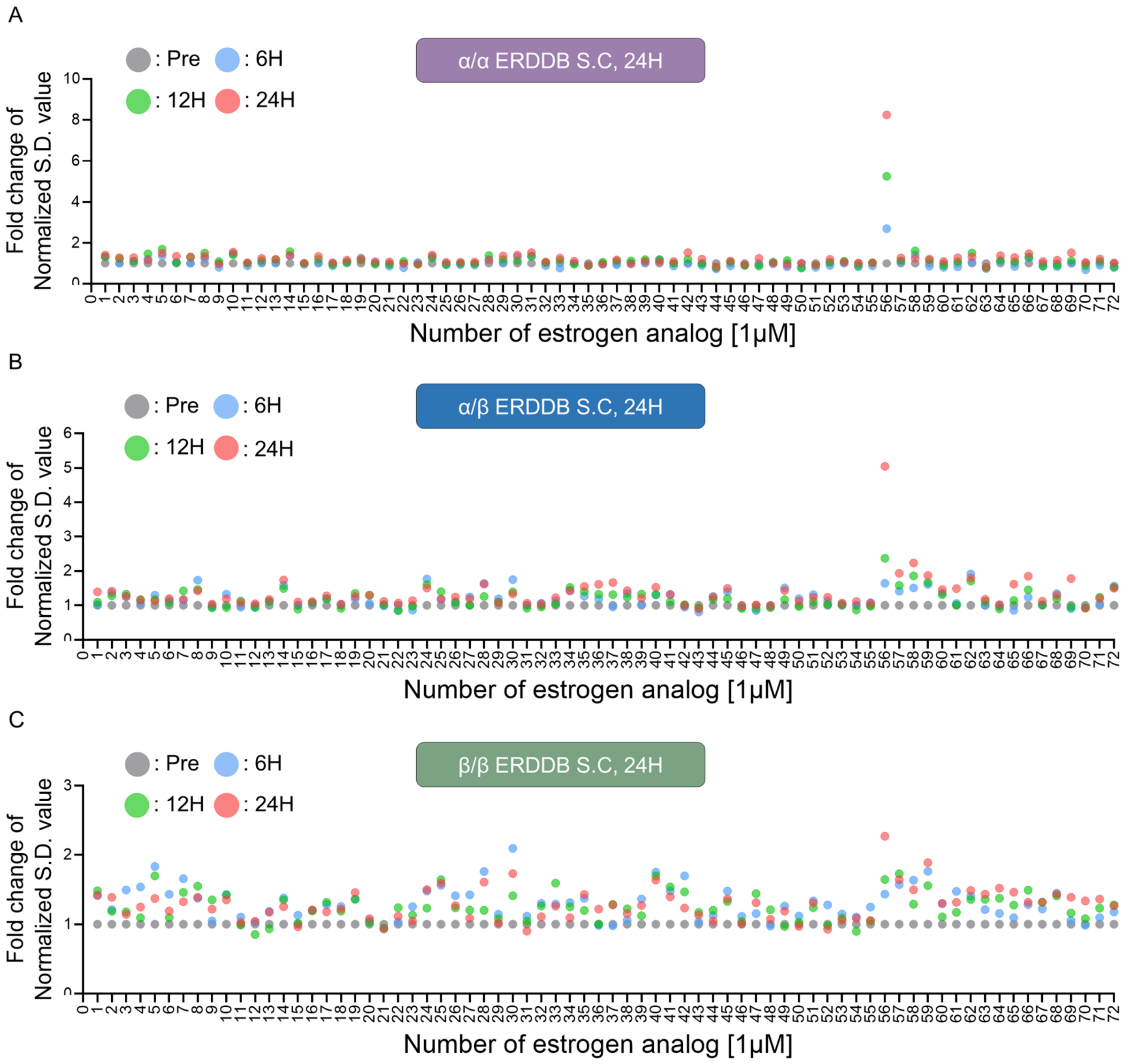

Figure S4. Standard deviation values of BRET ratio according to time after EA treatments

Standard deviation values of BRET ratio after 72 EA treatments in (A) α/α (B) α/β, and (C) β/β ERDDB S.Cs. Pre means before EA treatments.
